# A New Application for the Optimal Foraging Theory: The Extraction of Medicinal Plants

**DOI:** 10.1155/2012/364564

**Published:** 2011-09-22

**Authors:** Gustavo Taboada Soldati, Ulysses Paulino de Albuquerque

**Affiliations:** ^1^Botany Post-Graduation Program, Universidade Federal Rural de Pernambuco, Dom Manoel de Medeiros Street, s/n, Dois Irmãos 52171-900 Recife, PE, Brazil; ^2^Applied Ethnobotany Laboratory and Biology Department, Universidade Federal Rural de Pernambuco, Dom Manoel de Medeiros Street, s/n, Dois Irmãos, 52171-900 Recife, PE, Brazil

## Abstract

The Optimal Foraging Theory was used to identify possible patterns in bark extraction and the selective cutting of *Anadenanthera colubrina* (Angico), a medicinal plant. The hypotheses were built on two approaches: selection of collection place and bark exploitation occurrence in only one of these resource areas. The results suggest that the distance that must be traveled to reach each gathering site determines the extent of the extraction process, showing that people minimize the time and energy spent in *A. colubrina* collection. The availability of each site appears not to influence the operation. The resource amount was the optimized variable for bark extraction, which was analyzed in only one collection zone. In contrast to the phenomenon of collection place selection, the distance between angico individuals, the management period, and the tannin content did not affect bark extraction. This study also discusses how certain cultural aspects influence the extraction of angico.

## 1. Introduction

Ecological and evolutionary models are used in different studies on human populations and are important in trying to better understand the criteria for decisions concerning natural resource use and how human beings occupy living space because they allow for detecting patterns and predicting situations [1]. Despite being subject to some criticism [1–3], one of the most frequently used tools in human population studies is the Optimal Foraging Theory. The theory assumes that there is a cost/benefit ratio in obtaining any resource necessary to life and that natural selection favors foraging behavior, which optimizes species fitness [4, 5]. The energetic return (ratio of gained and spent energy in foraging events) is certainly the most studied variable in studies of optimal foraging, possibly due to the direct influence of researches in nonhuman animals. However, the Optimal Foraging Theory suggests that other variables are considered in the events of search and collection of resources [6], such as nutritional value.

There are different types of optimization within the theory that differ according to the biological process addressed, (see [5, 7–10]). MacArthur and Pianka [7] constructed a theoretical model in an attempt to determine which habitat (i.e., resource zone) species should visit during foraging activities because resources will vary with regard to their quality, abundance, and spatial distribution [7, 11]. 

This model predicts that more productive environments with higher resource biomass (resource availability) generally accumulate more consumers [6]. Thus, when the model of MacArthur and Pianka [7] is transposed to the reality of ethnobotanical research, it is expected that most productive areas with higher availability of plant environmental resources will have a larger amount of extraction events and will be more recognized locally, receiving, therefore, an increased amount of use citation. 

According to MacArthur and Pianka [7], foragers spend energy and time in obtaining resources. Therefore, the distance to be traveled to obtain a resource determines the choice of the site to be visited. It is expected that closer resource areas receive greater attention in plant resource collection and are subject to a greater number of extraction events. Additionally, the model considers that resource acquisition is divided into two distinct moments: the search itself and its management. Specifically for management, resources that are difficult to handle demand more time and energy [11]. In the case of ethnobotany, this means that, due to certain characteristics such as the presence of thorns and the hardness of wood or bark, resources that are more difficult to extract potentially have a lower preference of use.

Based on the foregoing research, the present study aimed to evaluate whether the use of plant resources in a rural community of Pernambuco follows the cost/benefit predictions of the Optimal Foraging Theory, focusing on the knowledge and the use of *Anadenanthera colubrina*, an important species to local communities in the Caatinga. The study was performed on two levels. The first approach is regional and aims to understand the selection of sampling sites to be visited during species collection, with the hypothesis that extraction events of *A. colubrina* are dependent on its density and the distance of these areas from the community. Additionally, it was tested whether the areas with the highest number of use citations are those with the highest densities of *A. colubrina* determined. The second approach focused on the bark harvested in one of the site collections, since that plant part is locally the most important. For this second approach, it was hypothesized that bark collection (total extraction events) is dependent on the distance to be traveled, bark thickness, difficulty of collection, and, finally, tannin content (resource quality). 

As previously pointed, the Optimal Foraging Theory considers other variables capable to optimization beyond energy. In this regard, transposing the optimal approaches to the use of medicinal plants, we hypothesized that the bark collection depends on the resource quality, in this case the amount of tannin provided by the resource. Therefore, both the bark thickness and its tannin concentration are important factors in this model.

Based on the results of this work, this study contributes to optimization theory in four ways. This was the first effort to empirically understand plant resource use under optimal foraging patterns within the Brazilian's semiarid context. Second, this study analyzed the extraction of a resource that is mainly used for medicinal purposes. Third, this study investigates both the harvested amount and the harvested resource quality of the species. Finally, the current research contributes to theory by discussing how biological nature and cultural systems interact to influence human behaviors.

## 2. Material and Methods

### 2.1. Study Area

The study was conducted in the municipality of Altinho (8° 29′ 32′′ S e 36° 03′ 03′′ W), which is located in the Agreste of Pernambuco, Northeast Brazil, characterized by a semi-arid warm climate (BSh) according to the Köppen classification, with average temperature around 23°C and annual rainfall between 300–1200 mm/year [12]. Altinho is located 160 kilometers (km) from the state capital and its population is approximately 22,000 people, evenly distributed between urban and rural areas [12]. The region is covered by a vegetal formation known as hipoxerophyte Caatinga, which is highly seasonal, deciduous, thorned and made up of different physiognomic and floristic compositions.

The research was performed in the Carão community (S 8° 35′ 225′′ and W 36° 05′ 576,′′ see Alencar et al. [13]), which is composed mainly of rural farmers who specialize in the production of maize, beans and palm. According to figures from the health center, 189 people live in the community, of which 112 are over age 18 (67 women and 45 men). The community is 16 km from the urban center of Altinho, with difficult access via non-paved roads [14].

The Carão community is located near a mountain known as the Serra do Letreiro, with a maximum altitude of 700 meters (m). It presents as a large mosaic of different seral stages in which shrubs and trees predominate. In relatively inaccessible areas, on the slope of the Serra do Letreiro, that, according to residents, have never suffered human intervention, there are plant communities in advanced succession stages. In other localities of the slope vegetal transformation has taken place in private areas for cultivation, especially of maize and beans. The mountain is an important source of plant resources, especially trees, for building or medical purposes. Other units that make up the local landscape and provide different resources include: the base of the slope that correspond to the mountain base, usually composed of several plant communities in initial succession stages triggered by the abandonment of pastures or crops, and the terrains, which is a particular and large area surrounding a residence, usually enclosed by fences. The terrains are composed of pastures, areas intended for grazing of animals where certain tree species with local importance, such as *Spondias tuberosa* Arruda and *A. colubrina*, are tolerated; manioc fields or planting, areas for cultivation of maize, beans and palm; and home gardens, areas immediately adjacent to the residences [15].

One of the most important plants used by Caatinga communities is the Angico* Anadenanthera colubrina* Vell. (Brenan) (Mimosaceae). This species plays important roles in different-use categories [16], although it is mainly used for traditional medicine [17]. In the community of Carão, the Angico is well known by the dwellers. The bark is mainly used for therapeutic purposes, and, because the medicinal category is considered the most important, the bark is the most exploited resource in the community. The bark is also exploited for its application to tanning (i.e., an artisanal processing of animal skins into leather); however, this activity is not common among the dwellers in Carão, and only two people are tanners within the community. Both the therapeutic and tanning properties result from the tannin within the species' bark. The use of Angico is strictly local, and only a few leather pieces are sold to the people of the region. There is no a regular commercial activity associated with Angico extraction.

### 2.2. Ethnobotanical Survey

This study is part of an ethnobotanical research program carried out since 2006 in the Carão community [13, 14, 18, 19]. Following the determinations in Resolution 196/96 of the National Council of Ethics, which governs research dealing with human beings, all informants who agreed to participate in the study signed a Term of Free and Informed Consent (FICT).

Information on the knowledge and use of *A. colubrina* necessary for testing the hypotheses was collected from semi-structured interviews [20] conducted between August 2006 and July 2007. A total of 36 men and 65 women aged 18 years or older, totaling 101 participants or 90.2% of the community's adult population, were interviewed. To access all known and used plants, a free listing technique was employed [20], in which the dwellers were asked to record the known plants and their used parts, attributed uses, collection places, and use forms. Only the information about Angico was used in this study. Information about the knowledge and use of the species will be discussed in a subsequent publication. The total number of citations in each action area was used to test whether the environmental availability (absolute density of *A. colubrina*) influenced the informants' consensus concerning the collection sites.

Subsequently, 15 residents were selected from among the participants who recognized *A. colubrina* as a resource in the initial interview to participate in a second stage of interviews. This phase aimed to explore more deeply the process of choosing and defining action areas to be visited for Angico extraction and the features observed in *A. colubrina* individuals making them suitable for bark collection. Among the 15 participants in this second phase of interviews were local experts (people recognized by their peers as holding considerable knowledge on the use of plants) on, in this case, *A. colubrina*. In this sense, the ethnobotanical information required for statistical tests and results discussion were collected from a triangulation of methods, using a quantitative and qualitative approach.

### 2.3. Availability and Extraction of *Anadenanthera colubrina*

**Figure 1:**
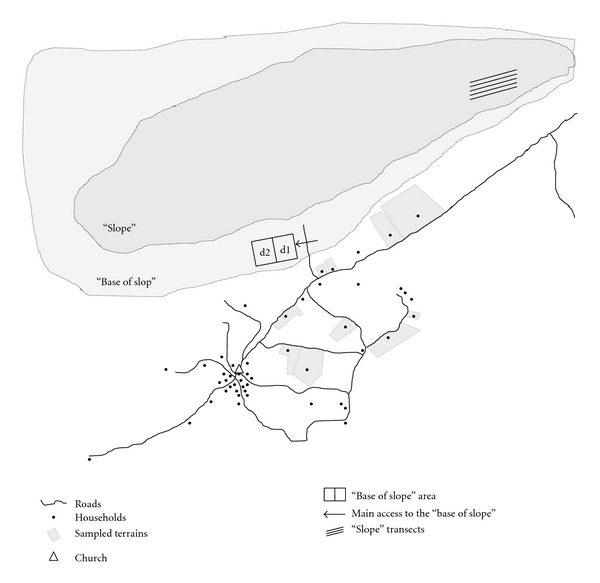
Sketch of the Carão community, Altinho municipality, PE, Brazil, showing the areas used to estimate the environmental availability of *Anadenanthera colubrina* (Vell.) Brenan. d1 and d2 represent the subdivision of base of slope into two blocks of distance used in the analysis of distance effect on the tannin concentration and Angico extraction.

To understand the selection of resource areas, it is considered the local classification of the landscape that was presented in detail by Almeida et al. [21]. For the present study, only three were considered, since they are the only areas recognized as a source of *A. colubrina: *slope, base of slope, and ground (Figure 1). Of these, only ground is not presented by Almeida et al. [21] and corresponds to regions adjacent to houses, for planting of fruit trees and small livestock. A more detailed map of the region is available in Alencar et al. [13].

Two different strategies were used for sampling vegetation; the choice depended on the characteristics of each area. In the slope, which has been little altered by humans according to the residents, the point-centered quarter method was used [22]. For this area, 180 quarters points were allocated at 20 m from each other. These points were equally divided into 9 parallel transects, which were also at 20 m distance from each other. This relatively inaccessible resource zone is located approximately 3 km from the core population. In the base of slope, a site with clear signs of bark extraction was chosen, with selective cutting where an *A. colubrina* population had grown. As this location was formerly a pasture and has well-defined boundaries, all of the Angico individuals present in a 2.7-hectare (ha) area were sampled. This population is located about 900 m from the community center. Finally, ten properties were selected for ground analysis and, as performed at the “base of slope,” had their area recorded and all Angico individuals sampled. 

Although distinct, it is believed that the sampling strategies used did not hamper or distort the data analysis. Firstly, these strategies estimate the same ecological parameter, the absolute density (AD), which was the variable used for tests in this study. Secondly, sampling at the base of slope and the grounds were the same, the only difference is that in this last collection area, the total area is subdivided among the properties visited. Finally, in addition to the sampling described for the slope, which considered an inclusion criterion, additional hikes were performed in the area to identify individuals of *A. colubrina*.

The CGL (circumference at ground level) was recorded for all *A. colubrina* individuals as well as the presence or absence of bark extraction or selective cutting events. The absolute density (number of individuals per hectare) was calculated according to the method of Araújo and Ferraz [22]. Such sampling information was used to test whether the Angico environmental availability in each gathering site determines the absolute and relative total number of exploitation events. To test whether the bark extraction is dependent on individuals' distance from the community, tannin content, bark thickness, and sampling difficulty, only the population sampled in the mountain range basis was considered (Figure 1). The choice of this area was based on the high density of Angico individuals, its presence in one of the locally recognized resource areas, its status as a common use area, the clear presence of sampling events and, finally, the fact that only one individual of *A. colubrina* had been sampled in the slope (which forbids the modeling proposed here). In this area, the individuals with diameter at breast height (DBH) larger than 6 cm had four bark samples obtained from different positions on the stem at a height of 2 m to determine the bark thickness, an indirect measure of the resource amount offered by each Angico individual and its tannin concentration. Bark thickness was estimated in the field using a caliper with 0.005-milimeter (mm) accuracy, and bark samples were then dried in an oven in the field. These dried bark samples were used for tannin concentration analysis. Finally, all individuals in the area were categorized into two types: few aculei (FA-type), when few and small aculei were present, and many aculei (MA-type), when they were large and densely distributed near the stem. In this sense, this classification aims to evaluate whether the difficulty of collection influences extraction events. Samples from different individuals of *A. colubrina* were collected and deposited into in the Professor Vasconcelos Sobrinho Herbarium (PEUFR), Federal Rural University of Pernambuco.

### 2.4. Tannin Concentration

Assuming that *A. colubrina* is important for its medicinal uses and for tanning animal leather due to the presence of tannins in its bark, the tannin concentration (grams per micro liter—g/*μ*L) was assessed as one of the variables to be optimized in the bark extraction process as an indirect measure of the resource quality. An adaptation of the Radial Diffusion Method [23] was used to determine the tannin concentration [24]. For sampling design, three factors were considered. First, the diameter classes were considered to investigate whether there is a relationship between concentration and individual diameter. The diameter at ground level (DGL) was measured for all individuals in the area, which is a standard methodology for Caatinga [25]. They were categorized into diameter classes with a 3-centimeter amplitude. These classes allow for analysis of the size and/or age phase of the population's individuals. The following classes were considered: 2 (3–5.99 centimeter-cm), 3 (6–8.99 cm), 4 (9–11.99 cm), 5 (12–14.99 cm), 6 (15–17.99 cm), 7 (18–20.99 cm), 8 (21–23.99 cm), 9 (24–26.99 cm), and 10 (27–29.99 cm). Second, aculeus density in the stem of each individual was considered according to the two types, FA-type and MA-type, which were previously described in Section 2.3. Finally, the last variable to be considered in the design was distance of the individuals with regard to ease of access. Thus, to determine whether the tannin concentration varied with distance, the area was subdivided into two blocks of 120 × 130 m, the first (d1) being nearer and the second (d2) more distant from the community (Figure 1). In this sense, the sample design to determine the tannin concentration was a 7 × 2 × 2 randomized block factorial design with three replications. Eighty-four individuals were randomly selected for tannin analysis.

### 2.5. Data Analysis

To assess whether there were differences in the amount of *A. colubrina* extraction between different resource areas, the absolute and relative total numbers of events of selective cutting and bark extraction were compared by chi-squared (contingency table). It is assumed a relationship between signal collection and use pressure in each area; that is, the use pressure in each area of collection is related to the frequency of use.

The slope was omitted from that analysis because it presented only one Angico individual, without evidence of exploitation. The distances between these areas and the community were estimated by GPS. The distance to be traveled to access each area was used as indirect measure of energy spent in collection events.

To assess whether there were differences between diameter classes in the bark extracted from the base of slope area, the total number of events in each class was compared using the *G* test. In addition to the chi-squared test, the *G* test was used to analyze data frequencies. A polynomial regression was performed to assess whether there is a link between diameter class and tannin concentration in the bark. This curve was used to analyze whether the diameter classes with higher frequencies of extraction events match the diameter classes with higher tannin concentrations. The total number of individuals with signs of bark extraction between the two types of Angico, the MA-type and the FA-type, was compared using the chi-squared contingency table analysis. Assuming that tannin concentration could vary between these two types of Angico, which, along with the gathering difficulties, could determine the intensity of bark extraction; the *t*-test was used to identify differences in tannins between these two types. Again, the use pressure is related to the total collection events.

*A. colubrina* individuals were categorized into six bark thickness classes with an interval of 1 cm, and the extraction events were compared by the *G*-test. As performed for tannin concentrations, a regression was performed to assess the relationship between diameter class and bark thickness. The resulting curve was used to examine whether the diameter classes with higher frequencies of collection correspond to those with greater bark thicknesses. Finally, to identify whether Angicos' distance in relation to the community influences the events of Angico collection, the categorization of the area into two blocks by distance, d1 and d2, was again considered. The extraction events between these two areas were also compared by the *G*-test. All tests were performed using statistical packages: Bioestat v 5.0 [26] and Startgraph v 5.1.

## 3. Results and Discussion

### 3.1. Resources Zone Selection

A total of 119 individuals of *A. colubrina* were sampled in the ground, 1040 in the base of slope and only one in the slope, corresponding to absolute densities of 4.33, 385.19, and 5.0 ind./ha, respectively, (Table 1). The greatest environmental availability of *A. colubrina* is, therefore, in the base of slope. Of the 119 Angicos existing in the ground area, 62 (52.1%) showed extraction signs, 18 (15.2%) of bark collection, and 48 (40.33%) of selective cutting. In the base of slope, 73 (7.01%) individuals were exploited, of which 34 (3.26%) showed signs of bark withdrawal and 42 (4.03%) of selective cutting. The single individual from the slope showed no evidence of exploitation. The total absolute (*X* ^2^ = 5.813, *P* = 0.0159) and relative (proportion of exploited to non-exploited individuals) (*X* ^2^ = 293.97, *P* = 0.0001) prevalence of bark extraction events and selective cutting were higher in the ground collection zone than those in the base of slope. 

These data suggest that the distance of resource areas from the community determines the choice of places to be visited in extraction events, with nearby zones visited most. In this sense, energy and time spent are minimized in collection events. Ladio and Lozada [27] studied the use of food plants by a community in Patagonia and tested different hypotheses related to the optimal use of these resources. Despite evaluating the full range of plants recognized as food, the authors found a similar situation, in which the community extracted a higher percentage of food resources in the vicinity of residences. The authors justify this pattern by the fact that two distant areas demanding a greater investment of time and energy and have a lower abundance of resources. Like the abundance of plants taken as food, studied by Ladio and Lozada [27], the environmental availability of *A. colubrina* does not define its extraction in the Carão community. Although the base of slope presents a significantly higher absolute density, this area does not have a higher density of bark extraction. Thus, it is possible to state that the distance from the resoursce area influences the selection of the site being visited; that is, the time and energy required to visit each area for the collection of *A. colubrina* are two variables to be optimized during extraction events.

Thus, the data suggest that the strategy used by the residents in the exploitation of *A. colubrina* is intended to reduce collection time. In other words, within the perspective of the Optimal Foraging Theory, residents optimize the return on events of Angico collection by reducing search time and, hence, the energy spent, rather than optimizing collection amount. This strategy may be a reflection of the main uses of *A. colubrina* in the production of medicinal infusions. For such use, only a relatively small volume of bark, about 200 g, is required.

The ground was cited 42 (60.86%) times in the interviews, the base of slope only once (1.44%), and the slope 26 (37.68%) times (Table 1). No differences were observed between the total citations of the two most cited sites (ground and slope), (*X* ^2^ = 0.0523, *P* = 0.0689). In this sense, the ground, areas closest to residences that suffer more use pressure, stands out in the number of citations received. The base of slope had a small number of citations attributed to it. However, in contrast to extraction data and despite being a more distant site with low abundance of *A. colubrina*, slope stood out in interviews in terms of the number of citations as much as ground. Unlike the collection events data, interview data suggest that the distance from the collection site and density of Angico do not determine residents' recognition of an area as a resource area. 

The interviews with the experts also pointed out some issues that are taken into account in the selection process for a resource area to be visited. The initial issue is that collection of plant resources such as Angico is not always independent of other activities. The residents affirmed that *A. colubrina* is collected when performing other tasks, such as herding cattle and weeding clearings. In this sense, collecting Angico in the mountains, where many areas of cultivation and grazing are located, is not a local preference but a fact because the interviewees spend much of their time devoted to their daily living activities. The residents also stated that *A. colubrina* is a rapid growth species, even after selective cutting. The local recognition of this growth warrants, in addition to proximity, the strong use pressure on individuals located in the ground. Additionally, there is a local belief that influences the choice of action areas. Some informants say that plants used for medicinal purposes cannot be collected from borders of roads or very frequented places because they are quite seen. Finally, another important issue is the political organization. Many residents reported not collecting *A. colubrina* in certain areas, especially in base of slope, because these are private properties.

The previously shown extraction and interview data suggest that *A. colubrina *extraction by the Carão community follows some premises of the Optimal Foraging Theory. However, some findings did not support this theory. How does an area like base of slope, with a density of *A. colubrina *almost ten times higher than the other areas, have only 7.01% of its Angicos exploited and go unrecognized as a resource area in the resident interviews? In conjunction with the gardens at each home, why was the slope the most cited and recognized resource area, despite its lower Angico density?

According to Martin [28], the collected information should be relative, there are difficulties in establishing what is good for humans because, in addition to their biological characteristics, social groups also respond to a cultural system. Accordingly, Sih and Milton [29] argued that the Optimal Foraging Theory should not be used simply to understand human behavior without a critical position. Therefore, this study reports that Angico extraction is driven by the interaction of environmental aspects, such as spatial distribution and resource density, with cultural factors of the Carão community. These cultural factors include spatial work distribution, dwellers' knowledge of species location on the slope, and resource collection.

Species use dynamics can also influence the selection of resource zones to be visited. The preferable use of the species is for medicinal purposes, and the current amount used is relatively low as use depends on illness events. Accordingly, collection does not compromise the Angico individuals or the dwellers because community members are aware of the species' spatial distribution and do not need to search for new individuals.

Therefore, as Martin [28] stated, the optimal outcomes in human populations occur within their cultural features. Future studies should be conducted to better understand this relationship as well as how cultural characteristics inherent to human groups reflect or distort the cost/benefit relationships in obtaining resources, especially in a longer time interval.

### 3.2. Bark Extraction in Base of Slope

#### 3.2.1. Collection Difficulty (Aculei)

As stated previously, 34 (3.26%) of 1040 *A. colubrina* individuals sampled in the base of slope presented evidence of bark extraction. Of all “Angicos,” 390 were classified as MA-type, with 14 (3.58%) being exploited, while 650 were categorized as FA-type, with 20 (3.08%) individuals presenting signs of collection. Despite interviews suggesting that the aculeus amount in the stem is a selection criterion, no differences in the proportions of exploited and nonexploited individuals for each type (*X* ^2^ = 0.199, *P* = 0.655) were observed. According to the informants, it is better to extract FA-type bark because it presents fewer aculei, its shaft is more rectilinear, it is easier to carry in large quantities, and its bark is more easily removed.

#### 3.2.2. Diameter Class and Bark Thickness

**Figure 2:**
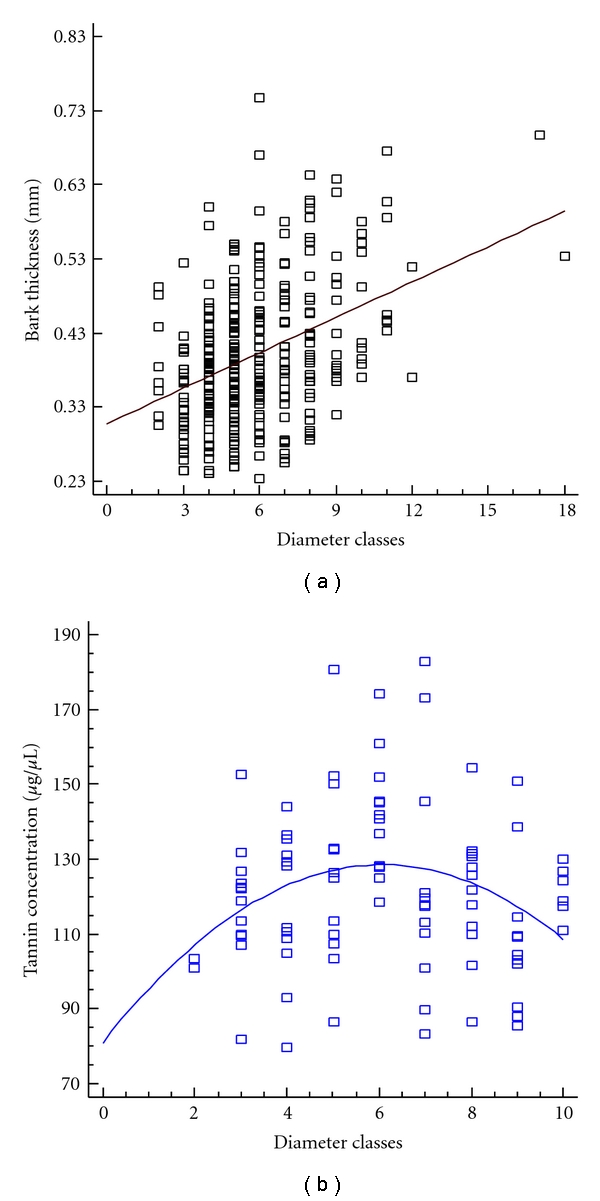
Tannin content and bark thickness in relation to the diameter classes in a population of *Anadenanthera colubrina* (Vell.) Brenan in the Carão community, Altinho, PE, Brazil. (a) Ratio between bark thickness and diameter classes from 2 (6–8,99 cm) to 18 (54–56,99 cm) (*Y* = 0.307286 + 0.0159271∗*X*; *P* = 0.001). (b) Distribution of tannin concentrations in relation to the diameter classes from 2 (6–8,99 cm) to 10 (30–32,99 cm) (*Y* = 80.7965 + 15.7183∗*X* − 1.29551∗*X*2, *P* = 0.0001).

When the total numbers of extraction events in each diameter class were compared, three classes stood out in terms of the number of exploited Angico: 8 (24–26,99 cm), 9 (27–29,99 cm), and 11 (33–35,99 cm) (Table 2). Thus, the bark extraction events are concentrated in the intermediate diameter classes.  Figure 2(a) shows how thickness relates to diameter classes, showing a linear and positive relation (*Y* = 0.307286 + 0.0159271∗*X*; *P* = 0.001; *R* ^2^ = 14.9575; *R* ^2^  adjusted = 0.1496; SD = 0.086; *F* = 58.04). The higher the diameter class, the greater the bark thickness, and; consequently, more resources are available for *A. colubrina* individuals. In this sense, because the larger diameter classes are represented by only four individuals, the data suggest that the bark extraction in the base of slope focuses on the greatest return of resources, concentrating on those individuals that provide a greater quantity (thickness) of bark. 

**Figure 3:**
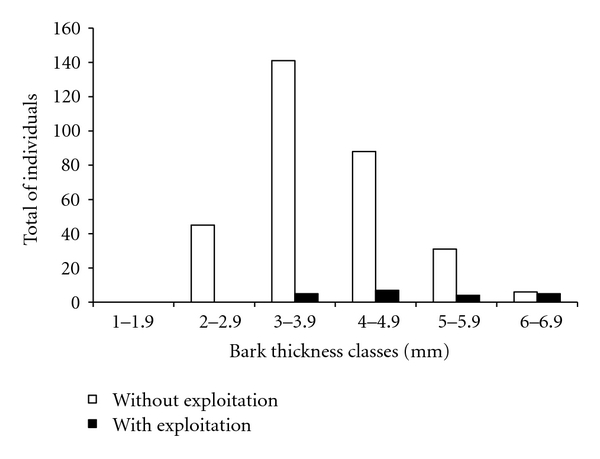
Bark extraction events of *Anadenanthera colubrina* (Vell.) Brenan divided into thickness classes in a population of base of slope, Carão community, Altinho, PE, Brazil.

Additionally, when Angico individuals in the area were categorized in relation to bark thickness and the total extraction events in these categories were compared, it appears that the largest category (thickness between 6.00 and 6.99 mm) is most exploited (*P* = 0.005) (Figure 3). Therefore, individuals with greater bark thickness were more heavily used during bark collection. This parameter is a criterion used when selecting the individuals of *A. colubrina* to be extracted.

#### 3.2.3. Tannin Concentration

The Angico individuals evaluated in the tannins test presented an average concentration of 121.46 g/*μ*L (SD = 21.90), with minimum and maximum concentrations of 79.0 and 183.0 g/*μ*L, respectively. Unlike the case of bark thickness, the diameter classes in which bark collection events are most frequent are not those with the highest tannin concentrations.  Figure 2(b) shows the relationship between diameter classes and tannin concentrations (*Y* = 80.7965 + 15.7183∗*X* − 1.29551∗*X* ^2^; *P* = 0.001; *R* ^2^ = 7.82496; *R* ^2^  adjusted = 0.0573; SD = 21.24; *F* = 3.72). The calculated polynomial curve shows that the tannin content increases with the diameter up to a peak and then decreases, and the concentration peak is found in individuals from diametric class 6 (18 to 20.99 cm). Therefore, the diameter classes that showed significant differences in the number of individuals exploited do not match the classes with the highest tannin content (tannin peak); that is, the classes with the greatest number of extraction events (8, 9 and 11) are in the range where tannin content decreases. Thus, content of tannin, the chemical compound responsible for the main uses of *A. colubrina*, is not optimized during bark collection in the area evaluated. 

Like the total number of individuals exploited in the two types, MA-type and FA-type, the tannin concentration did not differ between types (*P* = 0.4931). This information seems to be recognized by residents; despite the local preference to collect individuals of *A. colubrina* with few aculei, there is almost a consensus among them that the two types are equally strong.

#### 3.2.4. Individuals Distance

It was expected that Angicos nearer to the main access point of the base of slope area would present a greater number of collection events since their collection would demand less time and energy. However, there were no differences in the total number of individuals exploited between the two blocks of distance (*X* ^2^ = 0.436, *P* = 0.5093). Accordingly, bark collection is equally distributed in the base of slope and, therefore, is not influenced by the time and energy spent during extraction. 

Concerning bark collection specifically in the base of slope, the data suggest that there is no differential collection between the types few aculei (FA-type) and many aculei (MA-type). In this sense, as theoretical models suggest that easy management resources receive more attention, it was expected that the difficulty of collection would influence extraction. The models also predict that a difficult management resource will be exploited only if the return offsets the energy and time spent. As previously noted, unlike studies with food resources, which consider the return on energy as a measure of optimization, the tannin concentration was considered in the present study. However, as noted, tannin content also does not vary between the two morphological types of *A. colubrina.* The tannin content appears not to be a selection criterion for which Angico will have its bark exploited. The initial hypothesis was that, by conscious criteria or not, residents exploited barks of individuals with higher tannin levels. However, diameter classes with more extraction events did not correspond to those with the highest tannin levels. The classes with more exploitation did correspond, however, to those classes with the greatest bark thicknesses. Accordingly, the data indicate that, for bark collection, only the volume available for each Angico individual (bark thickness) is optimized because optimization based on tannin concentration does not occur. Such information may indicate that the resource quality for medicinal uses, measured by the tannin concentration, does not determine the collection and the optimization occuring by collecting larger bark volumes. The distance to be traveled between each individual and the main point of access to the area, an indirect measure of time and energy required, also did not influence the extraction. The area analyzed may be too small to assess the influence of distance or the existence of other access points.

## 4. Conclusion

This study shows that some aspects of *A. colubrina*, a primary medicinal resource; the extraction in the Carão community is influenced by environmental characteristics, as well as specific characteristics of individuals. At first, factors that determine the resource area selection to be visited during extraction events were analyzed. Subsequently, it was analyzed the factors that influence the specific house collection and medicinal resources more locally demanded at one of these collection areas. Both approaches are designed on the light of Optimal Foraging Theory, and the different variables analyzed in this study are summarized in Table 3. 

Few investigations have evaluated the use of plants based on the Optimal Foraging Theory [27, 30, 31], especially for medicinal plants. In this sense, the biggest difficulty was to transpose the optimum premises for medicinal uses. In principle, it is difficult to conceive an optimization process for collecting medicinal plants, since the foraging studies focus on food resources, traditionally having the energy as analytical factor. However, despite this theoretical basis be maintained when the distance of collection sites, accessibility, difficulty of collection, and amount collected were analyzed, given the specifics of the medical category, the collected energy was not considered as a variable to be optimized, but the tannin concentration. 

From this study it was concluded that the optimization ecological relationships can be verified in other use categories other than food and that, in principle, there are no theoretical limitations to the TFO use in different contexts of use. One of the major contributions of this research is that the data analyzed do not support the analogy between energy-mediated return (food crops) and bioactive compounds-mediated return (medicinal plants), that is, for optimum medicinal use seems not to consider the optimal plant quality, but the amount capable to be exploited.

The data from this study suggest that natural resource use, such as the extraction of *A. colubrina*, depends on both cultural and environmental factors and that human behavior is influenced by these two types of factors. Additionally, ecological theories and tools are important instruments for analyzing human populations. However, they need to be carefully used and further contextualized, especially when analyzing cultural specificities.

## Figures and Tables

**Table 1:** Comparison among the three areas known as collection sites of *Anadenanthera colubrina* (Vell.) Brenan in the Carão community, Altinho, PE, Brazil, emphasizing the ecological parameters and species extraction, as well as the recognition of areas in the interviews.

	“Grounds”	“Base of slope”	“Slope”
Total of sampled individuals	119	1040	1
Absolute density (ind./hectare)	4,33	385,19	5
Total of exploited individuals (%)	62 (52.1)	73 (7.01)	0 (0)
Bark extraction (%)	18 (15.2)	42 (4.03)	0 (0)
Selective cutting (%)	48 (40.33)	34 (3.26)	0 (0)
Citations in the interviews (%)	42 (60.86)	1 (1.44)	26 (37.68)

**Table 2:** Bark extraction of *Anadenanthera colubrina* (Vell.) Brenan by diameter classes and the types of many aculei and few aculei in a population of base of slope, Carão community, Altinho, PE, Brazil. *Diameter classes that concentrate extraction events in proportion.

Type of	“Many aculei” (MA)			“Few aculei” (PA)			All individuals		
diameter classes (cm)	Without signs	With signs	Total	Without signs	With signs	Total	Without signs	With signs	Total
1 (0–2.99)	15	0	15	216	0	216	231	0	231
2 (3–5.99)	69	0	69	142	1	143	211	1	212
3 (6–8.99)	86	0	86	103	0	103	189	0	189
4 (9–11.99)	66	2	68	58	1	59	124	3	127
5 (12–14.99)	51	1	52	51	2	53	102	3	105
6 (15–17.99)	33	2	35	24	2	26	57	4	61
7 (18–20.99)	16	1	17	16	4	20	32	5	37
8 (21–23.99)*	18	2	20	13	6	19	31	8	39
9 (24–26.99)*	8	1	9	4	2	6	12	3	15
10 (27–29.99)	10	0	10	1	1	2	11	1	12
11 (30–32.99)*	3	2	5	2	1	3	5	3	8
12 (33–35.99)	1	1	2	0	0	0	1	1	2
13 (36–38.99)	0	0	0	0	0	0	0	0	0
14 (39–41.99)	0	0	0	0	0	0	0	0	0
15 (42–44.99)	0	0	0	0	0	0	0	0	0
16 (45–47.99)	0	0	0	0	0	0	0	0	0
17 (48–50-99)	0	1	1	0	0	0	0	1	1
18 (51–53.99)	0	1	1	0	0	0	0	1	1

Total	376	14	390	630	20	650	1006	34	1040

**Table 3:** Summarization of the two approaches built from the Optimal Foraging Theory: resource zone selection and bark extraction in the base of the slope and the respective variables analyzed for the understanding of *Anadenanthera colubrina* (Vell.) Brenan extraction patterns in the community of Carão, Altinho, PE, Brazil.

Approach		
Resource zones selection	Extraction	Use citations
Availability of *A. colubrina *	No optimization	No optimization
Distance	Optimized	No optimization

Bark extraction in the base of slope	Extraction	Interviews

Tannin contration	No optimization	Optimized
Individuals distance	No optimization	Optimized
Diameter classes	Optimized	Optimized
Collection difficult (aculei)	No optimization	Optimized
